# Birth preparedness, complication readiness and other determinants of place of delivery among mothers in Goba District, Bale Zone, South East Ethiopia

**DOI:** 10.1186/s12884-016-0837-8

**Published:** 2016-04-06

**Authors:** Semere Sileshi Belda, Mulugeta Betre Gebremariam

**Affiliations:** Department of Public Health, College of Medicine and Health Sciences, Madawalabu University, P.O.Box 302, Bale-Goba, Ethiopia; Addis Ababa University, College of Health Sciences, School of Public Health, Addis Ababa, Ethiopia

## Abstract

**Background:**

Ethiopia is one of the countries with the highest maternal mortality ratio 676/100,000 LB and the lowest skilled delivery at birth (10 %) in 2011. Skilled delivery care and provision of emergency obstetric care prevents many of these deaths. Despite implementation of birth preparedness and complication readiness packages to antenatal care users since 2007 in the study area, yet an overwhelming proportion of births take place at home. The effect of birth preparedness and complication readiness on place of delivery is not well known and studied in this context.

**Methods:**

A community based case control study preceded by initial census was conducted on a total of 358 sampled respondents (119 cases and 239 controls) who were selected using stratified two stage sampling technique. A pre-tested and standardized questionnaire with a face-to-face interview was used to collect the data, and then data was cleaned, coded and entered in to SPSS version-21 for analysis. Binary logistic regression models were run to identify predictors of place of delivery and Odds ratio with 95 % CI was used to assess presence of associations at a 0.05 level of significance.

**Results:**

The mean (± Standard Deviation) age of respondents was; 27.41(±5.8) and 28.84(±5.7) years for the cases and the controls respectively. Two third (67.1 %) of the childbirths took place in the respondents house while only (32.9 %) gave birth in health facilities. Great proportion (79.7 %) of the cases and two third (34.0 %) of the controls were well-prepared for birth and complication. Maternal education, religion, distance from health facility, knowledge of availability of ambulance transport and history of obstetric complication were significantly associated with place of delivery (*P*-value <0.01). Birth preparedness and complication readiness practice had an independent effect on place of delivery (AOR =2.55, 95 % CI: 1.12, 5.84).

**Conclusion:**

The study identified better institutional delivery service utilization by mothers who were well-prepared for birth and complication. Strategies that increase the preparedness of mothers for birth and complication ahead of childbirth are recommended to improve institutional delivery service utilization.

**Electronic supplementary material:**

The online version of this article (doi:10.1186/s12884-016-0837-8) contains supplementary material, which is available to authorized users.

## Background

Every year eight million women suffer pregnancy-related complications and, almost the entire half million maternal deaths globally are in low- and middle-income countries. A woman in Sub-Saharan Africa (SSA) has a 1-in-22 chance of dying over her lifetime as a result of pregnancy, this risk is more than 200 times greater than the risk of a woman in the United States [1, 2]; yet the differential between high- and low-income countries remains higher than for any other health indicator [3]. Many of these deaths can be averted through skilled delivery care and provision of emergency obstetric care (EmOC) for women who develop complications [4].

The place of delivery (health institution or home) and the type of care a woman gets during childbirth determines the outcome of pregnancy. Skilled delivery care provided to women by a health professional within an enabling environment in a health centre or hospital is recommended to prevent maternal deaths [5]. Despite the national and global efforts; utilization rates of these services in rural Sub-Saharan Africa (SSA) with a skilled attendant (doctor, nurse, or midwife) is low; and the maternal mortality ratio (MMR) is declining sluggishly in the region [6].

Birth Preparedness and Complication Readiness (BPACR) is an overarching program approach to improve the use and effectiveness of key maternal and newborn health services, including skilled delivery service utilization based on the argument that preparing for birth and being ready for complications reduces all three phases of delays in receiving these services [7]. The lack of BPACR plan is one of the critical factors behind the sluggish progress towards the maternal target of the millennium development goals (MDGs). It is also considered as an intervention for preventive behaviour and programmatic approach to other socio-economic and cultural barriers which limit the fundamental gain from the health facilities [6]. Therefore BPACR at the individual women level; in terms of identifying a place for skilled delivery care, skilled birth attendant, saving money, arranging transportation, identifying emergency signs, identifying a health facility with 24 h emergency obstetric care and arranging blood donors [7], is crucial to improve the outcome of delivery by advance planning and preparation for delivery and complications.

Home deliveries are known to be associated with maternal morbidity and mortality, such as postpartum haemorrhage (PPH), birth trauma, infection, and fistula. Therefore women need timely access to skilled care during pregnancy, childbirth, and the postpartum period. Too often, however, their access to care is hampered by delays. The most important causes of these delays are common and predictable like transportation problem (71 %), followed by lack of money (68 %) and distance to a health facility (66 %) [8].

Ethiopia is one of the countries in SSA with the highest maternal mortality ratio (MMR) 676/100 000 live births in 2011 with no change from its previous level of 673/100,000 live births in 2005 [9]*.* Focused antenatal care (FANC) known in its preparation of the pregnant women to identify birth location and a skilled attendant is the approach used in Ethiopia and so in the Oromia regional state. Antenatal care from a skilled provider accounts to (33.9 %) nationally and (31.3 %) in Oromia region. Although during ANC pregnant women are encouraged to deliver in health institution that provide skilled delivery care, the utilization rates of skilled delivery service in 2011, remained low (10 %) in Ethiopia and (8 %) in the Oromia regional state where this study was conducted [8].

Even though Ethiopia has laid the foundation to improve maternal health with its strong commitment in policies, like the Health Extension Program (HEP) in reaching communities with basic health care and the implementation of BPACR packages to antenatal care (ANC) users since 2007 to encourage utilization of institutional delivery (ID) service [10], an overwhelming proportion of births continue to take place at home resulting in potentially preventable complications [8]. Meanwhile the concrete effect of birth preparedness and complication readiness on place of delivery and assistance by skilled birth attendants (SBA) is not well known and studied in this context. Thus, the aim of the study was to investigate the effects of maternal birth preparedness and complication readiness among the factors that determine place of delivery in Goba District, Oromia Regional State, South-Eastern Ethiopia.

## Methods

### Study area and period

The study was conducted from February 10^th^ to 5^th^ of March, 2014 in Goba District, which is one of the 18 Districts in Bale Zone, Oromia regional state, located at 444 Km in the South-East direction from the capital city Addis Ababa. Goba District is administratively structured into: Goba rural District and Goba administrative town, with 15 rural and 2 urban Kebeles (the smallest administrative unit with minimum of 1000 households) respectively. The estimated total population of the study district was 89,859 (projected from the 2007 census) [11]. There were an estimated 20,668 women of child bearing age (15-49 years) and 3774 pregnant women in the district during the study period. The public health infrastructure of the district involves; one referral hospital, four health centres and 15 functional health posts. The referral hospital in the town provides comprehensive emergency obstetric care while the health centres provide basic emergency obstetric care. The Health Posts are the lowest health facilities in Ethiopia used to improve equitable access to basic health services, mainly disease prevention and health promotion through a community (Kebele) based health extension program. Each health post is staffed by two female health extension workers (HEWs) who are assigned after completing one year of training. And the HEWs provide family planning, immunization, ANC, clean delivery service and postnatal care. Furthermore, they are responsible for referring women with obstetric complications to health centers and hospitals where basic and comprehensive emergency obstetric care is available.

### Study design

A community based unmatched case control study design; preceded by initial census i.e. to enumerate and prepare list of all households with eligible women in the sampled kebeles was used. The cases were mothers who gave childbirth in health institution and the controls were mothers who gave childbirth at home.

### Sample size and sampling technique

Childbearing women who gave childbirth in the last 12 months (February 10/2013 to February 09/ 2014) in the District, regardless of the birth outcome were included in the sample. The required sample size of eligible mothers for the study was determined with a formula to estimate two population proportion, using “*Epi-info7 Stat calc sample size for unmatched case control study”* with the assumptions of; a 95 % confidence level, 80 % power, level of exposure to BPACR package in the control group of 30.0 % [8], an expected odds ratio of 2.0, the ratio of controls to cases of 2:1, and an additional 5 % non-response rate. The calculated total sample size was 358 mothers who gave childbirth in the 12 months before the study period, with the number of sampled cases (*n* = 119) and sampled controls (*n* = 239).

Initially the study area was stratified in to rural and urban, and there were fifteen rural and two urban kebeles during the study time. Five rural and one urban Kebele were selected using lottery method. A house to house census of the sampled Kebeles was done and a total of 864 eligible households with women who gave childbirth from 10^th^ of February 2013 to 09^th^ of February 2014 (regardless of birth outcome) were identified and sample frames with a list of 285 eligible households with a mother who gave childbirth in a health facility and 579 eligible households with a mother who gave childbirth at home were prepared. From the selected six Kebeles, 119 households with women who gave childbirth in a health facility (cases) and 239 households with women who gave childbirth at home (controls) were selected by simple random sampling technique. If the households were locked or the mothers were not available at the time of data collection, frequent visits were made until the data collectors could communicate with them during the data collection period. A lottery method was used in cases where there was more than one eligible woman in a single household.

### Data collection instrument and procedures

Structured and pre-tested questionnaire was prepared first in English and then translated into Afan Oromo and Amaharic, local languages. Eight nurses had conducted the face to face interview and a health extension worker in each Kebele was used as a local guider during the preliminary census and the data collection periods. The principal investigator and two public health officers supervised the whole data collection process. Training was given to the data collectors and supervisors before the actual data collection regarding the aim of the study, data collection tool (going through each question), data handling, sampling procedure and interview techniques. The questionnaires were reviewed daily by the supervisors and the principal investigator to check for completeness and early corrections and cleaning of the data were made.

Data on place of delivery; on mothers’ age, religion, ethnicity, marital status, educational status, decision making of women on obstetric health care seeking, income, family size; on husbands age, education and occupation; availability of Television, Radio & Telephone in the household; on gravidity, parity, age at first delivery, number of abortions, still birth and live birth, obstetric complications experienced, antenatal care use, gestational age at the first ANC, the number (frequency) of ANC; awareness on availability and accessibility of health workers and skilled delivery care, service fee, knowledge of availability of free ambulance transportation service, average time of travel to the nearest health facility with emergency obstetric care; and on birth preparedness and complication readiness knowledge and practice of the mother were collected.

### Data processing and analysis

The data was checked visually for completeness, coded and entered into SPSS version 21 soft-ware package. Frequency distribution of the variables was used to check data entry errors and consistency was checked by doing double data entry on 10 % of the questionnaire. The results were presented in the form of tables, figures and texts. Frequencies and summary statistics such as mean, standard deviation and percentages used to describe the study population (cases and controls) in relation to relevant variables. Binary logistic regressions models were used to determine the effects of BPACR (the main exposure variable) and other covariates on the outcome variable (place of delivery). Variables were recruited for multivariable analysis based on findings from the bivariate analysis. To be a candidate for multiple logistic regressions; variables whose p-value < 0.25 along with the variables of known clinical importance were considered and we compared the coefficients of each variable with the coefficient from the model containing only that variable and tried to verify the importance of each variable in the multiple model using Wald statistic and variable that doesn’t appear to be important were eliminated, and new model were refitted until the important covariates were included. Furthermore lists of possible pairs of variables in the main effects model that have some scientific basis to interact with each other were formed. Then we added the interaction terms, one at a time, in the model containing all the main effects and then assessed its significance using the likelihood ratio test and dropped any non-significant interaction. Finally the overall goodness-of-fit of the Model was assessed using the Hosmer and lemeshow’s test. Adjusted Odds ratios and 95 % confidence intervals were computed for each explanatory variable to determine the strength of association with place of delivery while controlling the effect of potential confounders, at a *p*-value ≤ 0.05.

### Ethical considerations

Ethical approval was secured from designated institutional review committee of the Addis Ababa University, School of Public Health. A formal letter in request of cooperation was written to Goba District Health Offices. Information on the purpose of the study and the right not to participate were given to the participants and they were also informed that all data obtained from them would be kept confidential by using codes instead of any personal identifiers as presented in Additional file 1. Oral consent was obtained from each study participant. Information on the importance of getting services from skilled attendants during pregnancy, delivery and postnatal period were provided by the data collectors to the participants who delivered outside health institutions at the end of data collection. The study was adherent to the STROBE criteria as outlined in Additional file 2.

## Results

### Socio-demographic characteristics

One hundred eighty nine (53.1 %) of the respondents were from rural and 167 (46.9 %) of the respondents were from urban inhabitants. And about 203 (57 %) of the respondents were between the ages of 25 to 34 years, with mean (**±** standard deviation) age of 27.41 (±5.8) and 28.84 (±5.7) years for the cases and the controls respectively. Majority of the study participants 159 (44.7 %) were Muslim, and the other 149 (41.9 %) were Orthodox Christian and 48 (13.5 %) were Protestant by religion. Nearly three forth of the study participants 258 (72.5 %) were Oromo by ethnicity. Concerning educational background, 26 (22 %) of the cases and 146 (61.3 %) of the controls had no formal education, whereas 44 (37.3 %) of the cases and 21 (8.8 %) of the controls had secondary or post-secondary level education. Regarding marital status, 101 (85.6 %) of the cases and 220 (92.4 %) of the controls were in marital union.

Regarding husband’s education, 53 (44.9 %) of the cases and 28 (11.8 %) of the controls had secondary or post-secondary education, 34 (28.8 %) and 108 (45.4 %) of the cases and controls respectively had primary education. And on husband’s occupation status, 47 (39.8 %) of the case’s and 151 (63.4 %) of the control’s husbands were farmers. Eighty four (71.2 %) of the cases and 76 (31.9 %) of the controls travel less than half an hour to reach the nearest health facility (health centre or hospital) that provide emergency obstetrics care. And only few 6 (5.1 %) of the cases and 31 (13 %) of the controls reported the unavailability of any radio and/or television in their household, while 102 (86.4 %) and 159 (66.8 %) of the cases and the controls respectively reported the availability of a telephone or mobile phone in their household (Table 1).

**Table 1:** Socio demographic characteristics of the study participants (*n* = 356), Goba District, Bale Zone, South-East Ethiopia, February, 2014

Variables	Place of delivery		*P*-values*
	Cases (ID)	Controls (HD)	
	Frequency (%)	Frequency (%)	
Residence			
Urban	57(48.3)	110(46.2)	0.736
Rural	61(51.7)	128(53.8)	
Age of respondents			
15-19	7(5.9)	18(7.6)	0.021
20-24	36(30.5)	43(18.1)	
25-29	45(38.1)	82(34.5)	
30-34	16(13.6)	60(25.2)	
35-49	14(11.9)	35(14.7)	
Mean age (± SD)	27.41(±5.8)	28.84(±5.7)	
Religion of respondents			
Muslim	46(39.0)	113(47.5)	0.022
Orthodox	61(51.7)	88(37.0)	
Protestant	11(9.3)	37(15.5)	
Ethnicity of the respondents			
Oromo	76(64.4)	182(76.5)	0.002
Amahara	33(28.0)	30(12.6)	
Other (Tgrie, Guragie & Gamo)	9(7.6)	26(10.9)	
Marital status of respondents			
Married	101(85.6)	220(92.4)	0.109
Widowed/Divorced/Separated	11(9.3)	13(5.3)	
Not Ever Married	6(5.1)	5(2.1)	
Maternal (Respondent’s) Educational level			
No formal education	26(22.0)	146(61.3)	<0.001
1^0^ education	48(40.7)	71(29.8)	
2^0^ and above	44(37.3)	21(8.8)	
Occupation			
Housewife	75(63.6)	195(81.9)	<0.001
Merchants	19(16.1)	26(10.9)	
Employed (Government/Private)	20(16.9)	7(2.9)	
Other	4(3.4)	10(4.2)	
Monthly income of the women in Ethiopian Birr (ETB)			
None	56(47.5)	83(34.9)	0.130
None – 500	19(16.1)	49(20.6)	
501-999	17(14.4)	48(20.2)	
≥ 1000	26(22.0)	58(24.4)	
Total family income in Ethiopian Birr (ETB)			
< 500	20(16.9)	42(17.6)	0.177
500-1000	26(24.6)	81(34.0)	
> = 1001	69(58.5)	115(48.3)	
Family size			
< = 4	77(65.3)	93(39.1)	<0.001
5-6	27(22.9)	80(33.6)	
> = 7	14(11.9)	65(27.3)	
Husbands age (*n* = 327)			
< 30 years	30(25.4)	43(18.1)	0.125
> = 30 years	76(64.4)	178(74.8)	
Husbands educational level (*n* = 327)			
No formal education	19(16.1)	85(35.7)	<0.001
1^0^ education	34(28.8)	108(45.4)	
2^0^ and above	53(44.9)	28(11.8)	
Husbands’ occupational status(*n* = 327)			
Farming	47(39.8)	151(63.4)	<0.001
Employed (Government/Private)	33(28.0)	29(12.2)	
Merchant	18(15.3)	10(4.2)	
Other	8(6.8)	31(13.0)	
Type of Mass Media (working)			
Radio Working	54(45.8)	158(66.4)	<0.001
Television Working	58(49.2)	49(20.6)	
None	6(5.1)	31(13)	
Availability of Telephone (Mobile)			
Yes	102(86.4)	159(66.8)	<0.001
No	16(13.6)	79(33.3)	
Time spent (home to the nearest HI)			
< =30 minutes	84(71.2)	76(31.9)	<0.001
> 30 minutes	34(28.8)	162(68.1)	

*p-values were derived from a chi-square test

### Obstetric characteristics and reproductive health service use

Among the mothers interviewed nearly one third (33.9 %) of the cases and 29 (12.2 %) of the controls were primi-para mothers and 57 (48.3 %) of the cases and 151 (63.4 %) of the controls had their first pregnancy before the age of 20 years. Both the cases and the controls had nearly similar history of still birth, 9.3 % and 8.4 % respectively.

Among the respondents with ANC follow up; 68 (62.4 %) of the cases and 48(28.1 %) of the controls had the WHO recommended 4 or more ANC visits during the most recent pregnancy, and only 18 (16.5 %) of the cases and 11 (6.4 %) of the controls made the first ANC visit by skilled provider in the 1^st^ trimester. Concerning the decision maker for obstetric health service use in the household; 35 (29.7 %) of the cases and only 21 (8.8 %) of the controls make the decision by themselves, while 66 (55.9 %) of the cases and 163 (68.5 %) of the controls make the decisions jointly with their husband, and the other 17 (14.4 %) of the cases and 54 (22.7 %) of the controls reported that it is another person (husband/relatives) that makes the decision to seek obstetric health care (Table 2). Place of the recent childbirth from the preliminary census determined that; two third (67.1 %) of the childbirths in the 12 months before the study period were took place at home or outside health institution. Only (17.2 %) the rural mothers delivered the last child in health institution while (57.2 %) of the urban deliveries took place in health institutions.

**Table 2:** Obstetrics characteristics and service use of the study participants (*n* = 356), Goba District, Bale Zone, South-East Ethiopia, February, 2014

Variable	Place of delivery		*P*-Value*
	ID (Cases)	HD (Controls)	
	Frequency (%)	Frequency (%)	
Gravidity (number of pregnancies)			
1	40(33.9)	29(12.2)	<0.001
2-3	50(42.4)	86(36.1)	
≥ 4	28(23.7)	123(51.7)	
Parity (birth order)			
1	42(35.6)	29(12.2)	<0.001
2-3	54(45.8)	102(42.9)	
≥ 4	22(18.6)	107(45.0)	
Age at first pregnancy			
< 20 years	57(48.3)	151(63.4)	0.006
≥ 20 years	61(51.7)	87(36.6)	
Number of live births			
1	151(63.4)	34(14.3)	<0.001
≥ 2	87(36.6)	204(85.7)	
Number of abortion			
0	101(85.6)	192(80.7)	0.252
≥ 1	17(14.4)	46(19.3)	
Number of still birth			
0	107(90.7)	218(91.6)	0.772
≥ 1	11(9.3)	20(8.4)	
ANC in the very latest pregnancy			
I had ANC	109(92.4)	171(71.8)	<0.001
I had No ANC	9(7.6)	67(28.2)	
Gestational age at 1^st^ ANC (*n* = 280)			
≤ 12 weeks	18(15.3)	11(4.6)	<0.001
13-24 weeks	72(61.0)	85(35.7)	
≥ 25 Weeks	19(16.1)	75(31.5)	
Number of ANC visits (*n* = 280)			
1-3 visits	41(34.7)	123(51.7)	<0.001
≥ 4 visits	68(57.6)	48(20.2)	
Obstetric complications encountered			
Yes	34(28.8)	24(10.1)	<0.001
No	84(71.2)	214(89.9)	
Decision maker for obstetric service seeking			
Self	35(29.7)	21(8.8)	<0.001
Others (Husband/Relatives)	17(14.4)	54(22.7)	
Me & Husband jointly	66(55.9)	163(68.5)	

*p-values were derived from a chi-square test

### Reasons for non-institutional (home) delivery

The claimed major reasons by the respondents who delivered their last child outside health facility were; 127 (54.2 %) their labour was smooth and short, 110 (46.2 %) previous home delivery was normal, 38 (16 %) lack of person accompanying them to the health facility, 32 (13.4 %) presence of TBA and 19 (8 %) getting closer attention from relatives in their house.

### Obstetric complications experienced

**Fig. 1:**
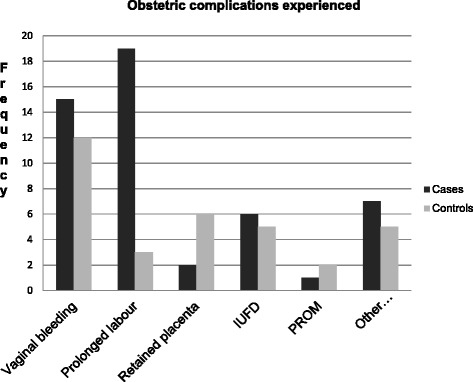
Types of complications faced by the study participants, Goba District, Bale Zone, South-East Ethiopia, February, 2014

About 58 (16.3 %) of the participants in general had a history of obstetric complication; out of them 34 (28.8 %) of the cases and 24 (10.1 %) of the controls reported that they had experienced some simple or serious form of complication during the most recent pregnancy, childbirth and/or postpartum period. Vaginal bleeding, prolonged labour, retained placenta, intra uterine fetal death (IUFD), loss of consciousness and premature rapture of membrane (PROM) were the common complications the respondents experienced (Fig. 1). All of the cases and 20 (83.3 %) of the controls who had complications received care from skilled provider for the complications.

### Birth preparedness and complication readiness knowledge

**Fig. 2:**
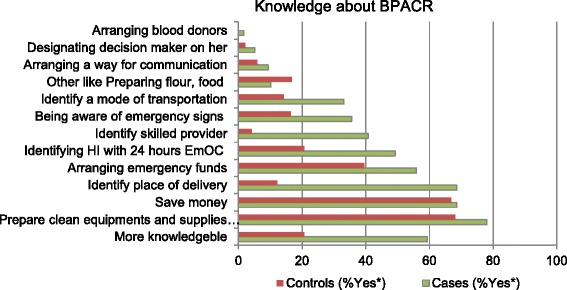
Place of recent delivery versus knowledge of preparation for birth and its complication Goba District, Bale Zone, South-East Ethiopia, February, 2014

On the assessment of the respondent’s knowledge about birth preparedness and complication readiness; 100 (84.7 %) of the cases and 108 (45.4 %) of the controls reported that they have ever heard the word “birth preparedness and complication readiness” (*P* < .0001). The majority of the mothers 134 (37.6 %) reported the source of information about the word BPACR is from health professionals, followed by health extension workers 103 (28.9 %), friends or family members 62 (17.4 %), and mass media 54 (15.2 %). Even though great majority 337 (94.7 %) of the study participants believe that a women needs preparation for normal birth and potential complications; the findings of their knowledge about BPACR revealed only 70 (59.3 %) of the cases and 49 (20.6 %) of the controls were knowledgeable (mentioned at least six of the BPACR component) (Fig. 2).

### Birth preparedness and complication readiness practice

This study used to construct a summary indicator of BPACR (arrangements) suggested by the JHPIEGO birth preparedness and complication readiness monitoring tool. Based on the seven arrangements (identified place of delivery or not, identified skilled birth attendant or not, saved money or not, identified means of emergency transport or not, arranged a blood donor for emergency or not, identified emergency signs or not and identified health institution with 24 h EmOC or not). The resultant BPACR index was used to examine the levels of BPACR practice among women. Women were categorized in to not well prepared (less than average preparation) and well prepared (more than average preparation).

Accordingly among the arrangements made ahead of the last delivery, 111 (94.1 %) of the cases and 89 (37.4 %) of the controls reported that they had identified a place for skilled delivery service, 87 (73.7 %) of the cases and 127 (53.4 %) of the controls reported they had saved money for emergency, only 9 (7.6 %) of the cases and none of the controls had arranged a blood donor for emergency. On aggregate, 94 (79.7 %) of the cases and 81 (34.0 %) of the controls arranged at least 4 out of seven (more than average) steps and hence were found well prepared for birth and complication ahead of last childbirth (Table 3).

**Table 3:** Number of BPACR arrangement steps taken by study participants ahead of last childbirth, Goba District, Bale Zone, South-East Ethiopia, February, 2014

Variables	Place of delivery	
	Cases(*n* = 118)	Controls(*n* = 238)
	Count (%)	Count (%)
Identified place of delivery	111(94.1)	89(37.4)
Identified skilled provider	73(61.9)	53(22.3)
Saved money	87(73.7)	127(53.4)
Identified means of emergency transport	101(85.6)	76(31.9)
Arranged a blood donor for emergency	9(7.6)	0(0.0)
Identified emergency signs	88(74.6)	127(53.4)
Identified HI with 24 hours EmOC	109(92.4)	171(71.8)
Number of steps (arrangements) taken	Count (%)	Count (%)
0	2(1.7)	35(14.7)
1	2(1.7)	45(18.9)
2	7(5.9)	44(18.5)
3	13(11.0)	33(13.9)
Well Prepared (> = 4 arrangements made)	94(79.7)	81(34.0)
Not well Prepared (<4 arrangements made)	24(20.3)	157(66.0)

### Knowledge of key obstetric danger signs

The most common type of key danger sign during pregnancy, childbirth and the postpartum period known by the respondents was vaginal bleeding; 86 (72.9 %) of the cases and 117 (49.2 %) of the controls knew vaginal bleeding is a key danger sign during pregnancy, 93 (78.8 %) of the case and 89 (37.4 %) knew excessive vaginal bleeding is a key danger sign during labour and delivery, and 71 (60.2 %) of the cases and 76 (31.9 %) of the controls knew excessive vaginal bleeding is a key danger sign during the post partum period. Only 26 (22 %) of the case and 26 (10.9 %) of the controls spontaneously reported difficulty of seeing or blurred vision is a key danger sign during pregnancy. Only 13 (11.0 %) of the case and 2 (0.8 %) of the controls spontaneously reported convulsion is a key danger sign during labour and delivery. And only 8 (6.8 %) of the case and 11 (4.6 %) of the controls knew increased body temperature (fever) is a key danger sign during the postpartum period (Table 4).

**Table 4:** Type of key obstetric danger signs spontaneously reported by respondents, Goba District, Bale Zone, South-East Ethiopia, February, 2014

Key obstetric danger signs	Place of delivery		*P*-values*
	Cases (*n* = 118)	Controls (*n* = 238)	
	Frequency (%)	Frequency (%)	
Key danger sign during pregnancy			
Vaginal bleeding	86(72.9)	117(49.2)	<0.001
Swelling of the face and hands	56(47.5)	35(14.7)	<0.001
Blurring (difficult) vision	26(22.0)	26(10.9)	0.005
Key danger sign during Labour and delivery			
Excess vaginal bleeding	93(78.8)	89(37.4)	<0.001
Prolonged labour (>12 hours)	76(64.4)	113(47.5)	0.003
Retained placenta(>30minuts)	54(45.8)	74(31.1)	0.007
Convulsions	13(11.0)	2(0.8)	<0.001
Key danger sign during postpartum period			
Vaginal bleeding	71(60.2)	76(31.9)	<0.001
Increased body temp (fever)	8(6.8)	11(4.6)	0.454
Offensive vaginal bleeding	21(17.8)	13(5.5)	<0.001

*p-values were derived from a chi-square test

### Factors associated with place of delivery

On Bivariate analysis; the educational of the mother, occupational status of the mother, the husband’s occupational and educational statuses, average time of travel from the respondents house to the nearby health facility with EmOC, family size, availability of television and telephone in the household, decision maker to get obstetric health care showed significant association with place of delivery. From the obstetric characteristics; gravidity, parity, age at first pregnancy, number of live births, mothers with obstetric complication, ANC visit during the last pregnancy, gestational age at first ANC, and number of ANC visits and among variables related to BPACR, knowledge and practice (status) of BPACR during the most recent pregnancy, knowledge of key obstetric danger signs during pregnancy, labour/delivery and postpartum period were the factors found to be significantly associated with place of delivery.

On multivariable logistic regressions analysis only maternal education, religion of the women, distance of home from the nearest health facility that provide EmOC, status of BPACR (number of BPACR steps/arrangements made), mothers who know the availability of free ambulance transport service and history of obstetric complication were found to be significantly associated with place of delivery (Table 5).

**Table 5:** Multivariate analysis of factors associated with place of delivery, Goba District, Bale Zone, South East Ethiopia, February, 2014

Variable	Place of delivery		COR (95 % CI)	AOR (95 % CI)
	Controls	Cases		
	Frequency (%)	Frequency (%)		
Respondent’s educational level				
No formal education	146(61.3)	26(22.0)	1	1
1^0^ education	71(29.8)	48(40.7)	3.80(2.18, 6.61)**	4.21(1.91, 9.32)**
2^0^ or post secondary	21(8.8)	44(37.3)	11.77(6.04, 22.91)**	3.40(1.15, 10.11)*
Religion of respondents				
Muslim	113(47.5)	46(39.0)	1	1
Orthodox	88(37.0)	61(51.7)	1.70(1.06, 2.73)*	1.29(0.62, 2.70)
Protestant	37(15.5)	11(9.3)	0.73(0.34, 1.55)	0.24(0.08, 0.70)**
Husband’s education (*n* = 327)				
No formal education	85(35.7)	19(16.1)	1	1
1^0^ education	108(45.4)	34(28.8)	1.41(0.75, 2.64)	0.42(0.17, 1.06)
2^0^ or post secondary	28(11.8)	53(44.9)	8.47(4.31, 16.65)**	0.62(0.19, 2.01)
Time spent (home to the nearest facility)				
< =30 minutes	76(31.9)	84(71.2)	5.27(3.25, 8.53)**	4.19(2.07, 8.49)**
> 30 minutes	162(68.1)	34(28.8)	1	1
Family size				
< = 4	93(39.1)	77(65.3)	1	1
> = 5	145(60.9)	41(34.7)	0.34(0.22, 0.54)**	0.55(0.26, 1.17)
Age at 1^st^ pregnancy				
< 20 years	151(63.4)	57(48.3)	1.86(1.19, 2.90)*	0.97(0.48, 1.95)
> = 20 years	87(36.6)	61(51.7)	1	1
Number of live births				
1	34(14.3)	42(35.6)	3.32(1.96, 5.60)*	1.49(0.59, 3.73)
> = 2	204(85.7)	76(64.4)	1	1
BPACR status				
Well Prepared	81(34.0)	94(79.7)	7.59(4.50, 12.80)**	2.55(1.12, 5.84)*
Not well prepared	157(66.0)	24(20.3)	1	1
Knew availability of free ambulance service				
Yes	33(13.9)	84(71.2)	15.35(8.92, 26.39)**	8.41(3.98, 17.79)**
No	205(86.1)	34(28.8)	1	1
Obstetric complication encountered				
Yes	24(10.1)	34(28.8)	3.61(2.02, 6.45)**	8.89(3.51, 22.52)**
^a^No	214(89.9)	84(71.2)	1	1

*p-value <0.05, **p-value <0.01, ^a^ includes “not sure”

Respondents with primary and secondary or post-secondary level of education were more likely to deliver in health institution than those mothers without formal education (AOR = 4.21, 95 % CI: 1.91, 9.32) and (AOR = 3.40, 95 % CI: 1.15, 10.11) respectively. Respondents of Protestant religion followers were found less likely to deliver in health institution as compared to Muslim religion followers (AOR = 0.24, 95 % CI: 0.08, 0.70). Respondents who access a health facility that provide EmOC with in less than 30 min travel are 4 times more likely to deliver in a health institution than mothers who travel more with (AOR = 4.19, 95 % CI: 2.07, 8.49).

Mothers who knew the presence of free ambulance transportation service for labouring mothers were more than 8 times more likely to deliver in health institution (AOR = 8.41, 95 % CI: 3.98, 17.79) than mothers who didn’t know the availability of the service. And mothers who had reported obstetric complications in the recent pregnancy and/or childbirth and/or postpartum period were more likely to deliver in health facility than mothers who hadn’t encountered complications (AOR = 8.89, 95 % CI: 3.51, 22.52). Those mothers who are well prepared for birth and complication (taken > =4 of the BPACR steps/arrangements) ahead of the last delivery were more than two and half times more likely to deliver in health institution (AOR = 2.55, 95 % CI: 1.12, 5.84) than those mothers who are not well prepared.

## Discussion

In this study we determined the utilization of health institutions that provide skilled delivery care for women during childbirth and examined the difference in birth preparedness, complication readiness and other characteristics of women who gave childbirth in a health institution and of those women who gave childbirth in their home during the most recent childbirth. Below, we discuss the main findings of the study.

### Birth preparedness and complication readiness of the women

This study revealed that birth preparedness and complication readiness status of the women ahead of childbirth is an important predictor of place of delivery. Those women who were well prepared for birth and complication were more than two and half times more likely to give childbirth in a health facility (health centre or hospital) than those women who were not well prepared. This finding also agrees with studies conducted in Nepal and Uganda where well prepared women were more likely seek location of skilled attendant for childbirth [12–14]. The study determined a better proportion of BPACR by women in the study area than BPACR by women in the Northern and Southern parts of Ethiopia. Our study revealed 80 % of the cases and 34 % of the controls were well prepared for birth and complications while 22 % of women in Adigrat town and 17 % of women in Aleta Wondo district of Northern and Southern Ethiopia respectively were well prepared for birth and complications [15, 16].

Even though well prepared mothers were found more likely to give birth in health facility; the study also identified that significant proportion (34 %) of well prepared women unfortunately delivered their babies at their home; this could be due to the local trends that mothers try the labour in their own house for a while and if it progresses smoothly they give birth in their home and they may not go to the health institution that provide skilled delivery care unless they identify problems in the labour and the home trial fails. And this could imply that though the mothers arrange the common resources made preparations for birth they are not using it until difficulties arise in labour and this could be due to the women’s inadequate knowledge to inspire the importance of institutional delivery or could be due to failure of the health professionals to establish trust and positive attitude by the community for health facility delivery even if the labour progresses without remarkable problem.

### Knowledge of key obstetric danger signs

The findings revealed that knowledge of the respondent about key obstetric danger signs was an important factor that determines the place of delivery during the last childbirth. Mothers who had better knowledge about key danger signs during pregnancy and childbirth were found more likely to deliver in health institution than those mothers who were less knowledgeable; and this finding is in line with reports from studies done in other developing countries [14, 17–20]. Further the result indicates that among those mothers who delivered at home (the controls groups) there is very low level of awareness about key danger signs and only 5 (2.1 %), 29 (12.2 %) and 4 (1.7 %) of the controls knew 3 key danger signs during pregnancy, childbirth and postpartum periods respectively. And also this is in line with findings reported by authors from Adigrat town and Robe Woreda, Ethiopia [13, 15].

One of the expected effects of knowledge about an issue is change on individual attitudes. Women who know danger signs or possibility of obstetric complications and the importance of a skilled provider and the care they provide tend to use delivery services by a skilled provider. The implications of this finding is that the need for community based programs or relevant intervention that provide information, education and communication on key danger signs during pregnancy, childbirth and postpartum periods; promote development of BPACR plan at individual, community and facility level; and that encourage the utilization of skilled delivery and ANC care by women, by targeting women who prefer non-skilled providers as well as improving the quality of care by providers will bring a positive contribution for utilizing skilled delivery care.

### Socio demographic characteristics

Our finding revealed that respondent’s educational status was found an independent predictor of place of delivery; respondents who had formal education were more than three times more likely to deliver in health facility; which was consistent with findings reported from the Northern and Southern parts of Ethiopia that indicated maternal education is a key determinant of skilled delivery service utilization [21–24]. There are a number of explanations that hypothesize as to why education is a key determinant of skilled care demand. For example education is likely to enhance female autonomy so that mothers develop greater confidence and capabilities to make decision regarding their own health, as well as their children. It is also more likely that educated women demand higher quality service and pay more attention to their health in order to insure better health for themselves. Moreover, this could imply that educated women are more likely to be aware of difficulties during pregnancy and as a result, they are more likely to use institutional delivery.

The study also revealed that the average time for travel from respondent’s household to the nearest health facility that provide emergency obstetric care (EmOC) is an independent predictors of place of delivery; women who travel less than 30 min were 4 times more likely to give birth in a health institution than women who travel more; which is in line with the study findings reported from elsewhere in Ethiopia and in other developing countries [20, 25]. This could be the result of living far from facilities that provide EmOC may disappoint the family or community’s support to a women in labour and women may fail to deliver in health institutions due to lack of community support or problems related to transportation access.

### Obstetric characteristics and service uses

Though ANC visit and number of ANC visits were not included in the multiple logistic regression models due to multi co-linearity effect with BPACR status of mothers; most of the obstetric variables including; gravidity, parity, age at first pregnancy, number of live births, ANC visit and number of ANC visits during the last pregnancy were significant association with place of delivery on Bivariate analysis which agrees with findings reported by other studies, that confirm obstetric factors and service uses like ANC were significantly associated with skilled delivery utilization [23, 25–27]. This could be due to the counselling services and other benefits mothers obtain from health workers during their ANC visits and previous experiences or benefits from health facility delivery.

Another important finding of the current study includes; mothers who know the availability of free ambulance transport service which is currently available for a women (pregnant mothers who are in labour and/or those who faced problems) and need an urgent transportation to a health institution that provide EmOC at any time. Mothers who knew the availability of free ambulance transport service in the district were eight times more likely to deliver in health institution than those mothers who do not know the availability of the service, and this finding is also in line with the study report from other studies [25]. This could be due to being aware the availability of the service can motivate the utilization of the service and it could imply the importance of providing free ambulance transport service can solve the transportation related challenges of the family and encourages the women and her family to use skilled delivery services with the help of the readily available transport service.

Obstetric complication during the recent pregnancy was another important determinant of place of delivery; women who faced problems (complications) related to the most recent pregnancy were nearly 9 times more likely to give birth at health facility than those respondents who had no obstetric complications in the most recent pregnancy. This agrees with results from other studies conducted in Sheka and Gondar zones in Ethiopia [21, 28]. This could imply that women who had a complication during pregnancy were more likely to visit a skilled provider for treatment of the pregnancy complication and they received counseling and strong recommendations on the importance of delivering in a health facility that provide a skilled care.

### Strengths and limitations of the study

The study used a preliminary census to identify and list eligible study subjects from whom cases and controls were selected and from the census we determined the actual magnitude of institutional delivery service utilization in the study area. And the data collectors worked jointly with health extension workers to maximize the response rate and possibly to reduce selection and information biases since they know all the localities of their respective Kebeles. For time and logistic reasons the study was limited to assess birth preparedness and complication readiness at the individual (mother’s) level, however assessing BPACR at family or community and health facility level would bring further evidence on the effects of BPACR at different level.

## Conclusions

Mother’s birth preparedness and complication readiness status ahead of childbirth had an independent effect on place of delivery. The study identified better institutional delivery service utilization among mothers who were well-prepared for birth and complication. The other factors associated with place of delivery in the study area includes; maternal education, distance of respondents home from the nearest health facility that provide emergency obstetric care, respondent’s knowledge about the availability of free ambulance transport service and history of obstetric complication during the most recent pregnancy.

While the respondent’s awareness about birth preparedness and complication readiness was relatively good; poor recognition of key obstetric danger signs by the respondents was identified in the study area. Therefore BPACR plan at individual, community and facility level are recommended and programs should give emphasis to information, education and communication on key danger signs during pregnancy, childbirth and postpartum periods.

Although skilled delivery service is physically available; mothers who were not well prepared for birth and complication and mothers who had no ANC care during pregnancy are less likely to use the services. Therefore programs and health services should promote the use of ANC by all women during pregnancy and inspire women the use of available skilled care for childbirth.

## Additional files

Additional file 1. Data collection instrument—English Version participant information sheet, consent and questionnaire. (DOCX 66.1 kb) Additional file 2. STROBE Statement—Checklist of items that should be included in reports of *case-control studies*. (DOC 92 kb)

## Abbreviations

ANC: antenatal care
AOR: adjusted odds ratio
BP: birth preparedness
BPACR: birth preparedness and complication readiness
BPP: birth preparedness program
COR: crude odds ratio
CSA: central statistical agency
EDHS: Ethiopian demographic and health survey
EmOC: emergency obstetrics care
ETB: Ethiopian birr
FANC: focused antenatal care
HD: home delivery
ICPD: international conference on population and development
ID: institutional delivery
IUFD: intra uterine fetal death
JHPIEGO: Johns Hopkins program for international education in gynaecology & obstetrics
MDG: millennium development goal
MMR: maternal mortality ratio
MNH: maternal and neonatal health
PNC: post natal care
PROM: premature rapture of membrane
SBA: skilled birth attendant
SPSS: statistical package for social science
SSA: Sub Sahara Africa
TBA: traditional birth attendant
TTBA: trained traditional birth attendant
UNFPA: United Nations fund for population agency
UNICEF: United Nations international children’s fund
WHO: World Health Organization

## References

[1] Organization WH. Beyond the Numbers: Reviewing maternal deaths and complications to make pregnancy safer. Geneva: World Health Organization; 2004.

[2] Organization WH, UNICEF. Maternal mortality in 2005: estimates developed by WHO, UNICEF, UNFPA, and the World Bank. 2007.

[3] Burchett HE, Mayhew SH (2009). Maternal mortality in low-income countries: What interventions have been evaluated and how should the evidence base be developed further?. Int J Gynecol Obstet.

[4] Campbell OM, Graham WJ (2006). Strategies for reducing maternal mortality: getting on with what works. Lancet.

[5] Safer MP (2004). Making pregnancy safer: the critical role of the skilled attendant.

[6] Lee AC, Lawn JE, Cousens S, Kumar V, Osrin D, Bhutta ZA, Wall SN, Nandakumar AK, Syed U, Darmstadt GL (2009). Linking families and facilities for care at birth: what works to avert intrapartum-related deaths?. Int J Gynecol Obstet.

[7] Roxana C, Del Barco, Ed. Monitoring Birth Preparedness and Complication Readiness. Baltimore: Tools and Indicators for Maternal and Newborn Health, JHPIEGO. 2004.

[8] Central Statistical Agency. Demographic B. Health Survey 2011. Addis Ababa, Ethiopia; 2013.

[9] Haub C (2006). Ethiopia Demographic and Health Survey 2005.

[10] Koblinsky M, Tain F, Gaym A, Karim A, Carnell M, Tesfaye S (2010). Responding to the maternal health care challenge: The Ethiopian Health Extension Program. Ethiop J Health Dev.

[11] United N. Federal Democratic Republic of Ethiopia. Summary and Statistical Report of the 2007 Ethiopian Population and Housing Census Results. Addis Ababa; 2008.

[12] Nawal D, Goli S (2013). Birth preparedness and its effect on place of delivery and post-natal check-ups in Nepal. PLoS One.

[13] Kaso M, Addisse M (2014). Birth preparedness and complication readiness in Robe Woreda, Arsi Zone, Oromia Region, Central Ethiopia: a cross-sectional study. Reprod Health.

[14] Kabakyenga JK, Östergren P-O, Turyakira E, Pettersson KO (2011). Knowledge of obstetric danger signs and birth preparedness practices among women in rural Uganda. Reprod Health.

[15] Hiluf M, Fantahun M (2008). Birth preparedness and complication readiness among women in Adigrat town, north Ethiopia. Ethiop J Health Dev.

[16] Hailu M, Gebremariam A, Alemseged F, Deribe K (2011). Birth preparedness and complication readiness among pregnant women in Southern Ethiopia. PLoS One.

[17] van de Ven R (2009). A qualitative study to explore why some women refrain from institutional delivery despite attending antenatal care in rural Malawi.

[18] Manandhar DS, Osrin D, Shrestha BP, Mesko N, Morrison J, Tumbahangphe KM, Tamang S, Thapa S, Shrestha D, Thapa B (2004). Effect of a participatory intervention with women’s groups on birth outcomes in Nepal: cluster-randomised controlled trial. Lancet.

[19] Karkee R, Lee AH, Binns CW (2013). Birth preparedness and skilled attendance at birth in Nepal: implications for achieving millennium development goal 5. Midwifery.

[20] Kabakyenga JK, Östergren P-O, Turyakira E, Pettersson KO (2012). Influence of birth preparedness, decision-making on location of birth and assistance by skilled birth attendants among women in south-western Uganda. PLoS One.

[21] Asres A, Davey G (2014). Factors associated with safe delivery service utilization among women in Sheka Zone. Southwest Ethiopia. Matern Child Health J..

[22] Worku AG, Yalew AW, Afework MF (2013). Factors affecting utilization of skilled maternal care in Northwest Ethiopia: a multilevel analysis. BMC Int Health Human Rights.

[23] Mengesha ZB, Biks GA, Ayele TA, Tessema GA, Koye DN (2013). Determinants of skilled attendance for delivery in Northwest Ethiopia: a community based nested case control study. BMC Public Health.

[24] Abebe F, Berhane Y, Girma B (2012). Factors associated with home delivery in Bahirdar, Ethiopia: A case control study. BMC Res Notes.

[25] Teferra AS, Alemu FM, Woldeyohannes SM (2012). Institutional delivery service utilization and associated factors among mothers who gave birth in the last 12 months in Sekela District, North West of Ethiopia: A community-based cross sectional study. BMC Pregnancy Childbirth.

[26] Tsegay Y, Gebrehiwot T, Goicolea I, Edin K, Lemma H, Sebastian MS (2013). Determinants of antenatal and delivery care utilization in Tigray region, Ethiopia: a cross-sectional study. Int J Equity Health.

[27] Amano A, Gebeyehu A, Birhanu Z (2012). Institutional delivery service utilization in Munisa Woreda, South East Ethiopia: a community based cross-sectional study. BMC Pregnancy Childbirth.

[28] Fikre AA, Demissie M (2012). Prevalence of institutional delivery and associated factors in Dodota Woreda (district), Oromia regional state, Ethiopia. Reprod Health.

