# Supplementary material for: Birth preparedness, complication readiness and other determinants of place of delivery among mothers in Goba District, Bale Zone, South East Ethiopia
Source: BMC Pregnancy Childbirth. 2016 Apr 6;16:73. doi: 10.1186/s12884-016-0837-8 (PMC4822318; doi:10.1186/s12884-016-0837-8)
Supplement: Additional file 1. — Data collection instrument—English Version participant information sheet, consent and questionnaire. (DOCX 66.1 kb) [file 12884_2016_837_MOESM1_ESM.docx]

**English Version participant information sheet, consent and questionnaire**

**Participant Information sheet and Verbal Consent form**

My name is. ----------------------------------------------- (Interviewer)

I am working with a graduate student researcher from Addis Ababa University, School of Public Health. This is a study to be conducted with the title “Birth preparedness, complication readiness and other determinants of place of delivery among mother in Goba District, Bale Zone, South East Ethiopia”. You are one of the women who have been selected randomly to participate in this study. Therefore, you are kindly requested to participate in this study and provide the information required from you. I would like to ask you a few questions if I may, but you have the right not to answer any question I ask. You may end the interview at any time. You can also refuse to participate in the study entirely. Your refusal will not restrict you from obtaining the required medical care when you need. The interview will last approximately 30-45 minutes. Your responses will be kept confidential and there will be no way of linking your individual responses to the final results of the study findings. I would like to inform you that the responses that you provide to the questions are very essential, not only for the successful accomplishment of the study, but also for producing relevant information which will be helpful in the planning and implementation of intervention to prevent delays, increase institutional delivery and use of skilled birth attendant.

Given all the above information, may I Proceed with the questions? (Mark “X” in the box)

Yes proceed with the interview; No thank her and End.

If you have any question, you can contact the following person with the address given here:

**Semere Sileshi** (Mob No 0911990843 or 0920682030) (Email: sem_sileshi@yahoo.com)

Name of interviewer: _________________ Signature ____________ Date: _______________

Name of supervisor: ________________Signature: _____________________

Questionnaire code --------------------- Kebele ---------------- House number------------------

**Questionnaire**

**Instruction**: - Circle the responses for questions with alternatives and write for open ended questions on the space provided.

**Section 1.Sociodemographic Information**

| S.no. | Questions | | | | Alternative responses | | | | | | | | | | Code | Skip |
| --- | --- | --- | --- | --- | --- | --- | --- | --- | --- | --- | --- | --- | --- | --- | --- | --- |
| 101 | What is your age? | | | | **________**Year | | | | | | | | | |  |  |
| 102 | To which religion do you belong? | | | | 1. Muslim 2. Orthodox 3. Protestant 4. Other | | | | | | | | | |  |  |
| 103 | What is your ethnicity? | | | | 1. Oromo 2. Amhara 3. Tigrae 4. Guragie 5. Other (specify)___________ | | | | | | | | | |  |  |
| 104 | What is your current marital status? | | | | 1. Married/in Union 2. Single 3. Widowed 4. Divorced 5. Separated | | | | | | | | | |  |  |
| 105 | What is the highest grade you completed? | | | | 1. None 2. Read and write 3. Primary 4. Secondary and above | | | | | | | | | |  |  |
| 106 | What is your occupation? | | | | 1. Housewife 2. Gov’t. employee 3. Private employee 4. Merchant 5. Other specify……….. | | | | | | | | | |  |  |
| 107 | Monthly income in Eth.Birr | | | | ______________Eth.birr. | | | | | | | | | |  |  |
|  | **If married or in union** | | | |  | | | | | | | | | |  |  |
| 108 | Age of husband in completed years | | | | | ___________years. | | | | | | | | |  |  |
| 109 | What is the highest grade your husband completed? | | | | 1. None 2. Read and write 3. Primary 4. Secondary and above | | | | | | | | | |  |  |
| 110 | What is your husband’s current occupation? | | | | | | | | | 1. Farming 2. Gov’t. employee 3. Private employee 4. Merchant 5. Other specify……… | | | | |  |  |
| 111 | Monthly income of your husband. | | | | | | | ______________Eth.birr | | | | | | |  |  |
| 112 | Total household income per month. | | | | | | | ______________Eth.birr | | | | | | |  |  |
| 113 | Which Mass media do you access in your house? (more than one option is possible) | | | | | | | 1. Radio working order 2. Television working order 3. Other specify ______ | | | | | | |  |  |
| 114 | Does any member of your house hold have a Telephone/Mobile Phone? | | | | | | | 1. Yes 2. No | | | | | | |  |  |
| 115 | Family size | | | | ……………………… | | | | | | | | | |  |  |
| 116 | How long it takes from your home to health center/hospital in minutes? | | | | 1. <15minutes 2. 15-30minutes 3. >30minutes | | | | | | | | | |  |  |
| 117 | Who is the decision maker for health service seeking during pregnancy, delivery and postpartum period? | | | | 1. Self 2. Husband 3. Self and Husband jointly 4. Other specify______________. | | | | | | | | | |  |  |
| **Section II: gravidity and parity/obstetric information.** | | | | | | | | | | | | | | | | |
| S.no. | Questions | | | Alternatives responses | | | | | | | | | | | Code | Skip |
| 201 | According to your birth order where does the last birth belongs? | | | 1. First 2. Second 3. Third 4. Fourth and above | | | | | | | | | | |  |  |
| 202 | How old were you in your **first** pregnancy? | | | ___________years | | | | | | | | | | |  |  |
| 203 | How old were you in your **last** pregnancy? | | | ___________years | | | | | | | | | | |  |  |
| 204 | How many times you became pregnant in your life? | | |  | | | | | | | | | | |  |  |
| 205 | What were the outcomes of the pregnancies? (Ask for each item and put numbers on the space for each event) | | | 1. Total lives birth……. 2. Abortion………. 3. Still birth……… 4. Others (specify)….. | | | | | | | | | | |  |  |
| **Section III: Service use and planning actions: intention and behaviors/knowledge** | | | | | | | | | | | | | | | | |
| S.no. | Questions | | | Alternatives responses | | | | | | | | | | | Code | Skip |
| 301 | Did you attend ANC before your last pregnancy? | | | 1. Yes 2. No 3. It was my first | | | | | | | | | | |  |  |
| 302 | Did you planned to attend ANC visits during your last pregnancy? | | | | | | 1. Yes 2. No | | | | | | | |  |  |
| 303 | Did you attended ANC visits during your last pregnancy? | | | 1. Yes 2. No | | | | | | | | | | |  | Q307 |
| 304 | If yes, Whom did you see during your last ANC visits? | | | 1. Physician 2. Health Officer 3. Nurse 4. HEWs 5. Others (specify) ------------------ | | | | | | | | | | |  |  |
| 305 | At how many weeks /months of pregnancy you start ANC? | | | ___________weeks/months. | | | | | | | | | | |  |  |
| 306 | How many times you attend ANC in last pregnancy? | | | 1. One 2. Two 3. Three times 4. Four and above | | | | | | | | | | |  |  |
| 307 | Have you ever heard the term “birth preparedness? | | | 1. Yes 2. No | | | | | | | | | | |  | Q309 |
| 308 | If yes; From whom did you get the information? **(more than one answer is possible)** | | | 1. Health professional 2. CHW /HAD or one-in-five 3. TTBA 4. Friends and relatives 5. Media: TV or Radio 6. Other(specify)_______________ | | | | | | | | | | |  |  |
| 309 | In your opinion, does a woman need preparation for birth? | | | 1. Yes 2. No | | | | | | | | | | |  | Q401 |
| 310 | In your opinion, what are some things a woman can do to prepare for birth?  (**more than one answer is possible)** | 1. Identify place of delivery 2. Save money 3. Prepare essential items for clean delivery & post partum period 4. Identify skilled provider 5. Being aware of the signs of an emergency & the need to act immediately 6. Designating decision maker on her 7. Arranging a way to communicate with a source of help 8. Arranging emergency funds 9. Identify a mode of transportation 10. Arranging blood donors 11. Identifying the nearest institution that has24 hours functioning EmOC services 12. Other (specify) --------------------------------------------- | | | | | | | | | | | | |  |  |
| **Section 4፡ Practices of respondents on preparation and readiness for birth and related problems** | | | | | | | | | | | | | | | | |
| **401** | Did you identify place of delivery for the last delivery? | | | 1. Yes 2. No | | | | | | | | | | |  | Q403 |
| 402 | If yes, Where was the place of delivery you planned? | | | 1. Home 2. Gov. hospital 3. Health center 4. Health post 5. Others specify | | | | | | | | | | |  |  |
| 403 | Where did you deliver your last child? | | | 1. Home 2. Health post 3. Gov. hospital 4. Health center 5. Others (specify)----------- | | | | | | | | | | |  | Q405 |
| 404 | If **home**, Why did you prefer to deliver at home?  (**More than one response is possible**) | | | 1. Too much cost of HFs 2. Facility too far 3. Poor quality service of HFs 4. No female provider at HFs 5. Husband will not allow 6. Need to be with relatives 7. Presence of TBAs 8. Labor was smooth and short 9. Previous HDs was normal 10. Lack of accompanies 11. I was told my pregnancy is normal 12. Lack of transport 13. Others specify. | | | | | | | | | | |  |  |
| 405 | If HFs, Why did you prefer to deliver in health facilities? (more than one answer is possible) | | 1. HF was near to me 2. Need better service 3. Previous better outcome with delivering at HF 4. I was told to deliver at health facilities 5. Difficult labor 6. Bad outcome with previous delivery 7. HF delivery is always needed 8. Other | | | | | | | | | | | |  |  |
| 406 | What was the mode of your last delivery? | | 1. Spontaneous vaginal delivery 2. Instrumental delivery 3. Cesarean section 4. I did not remember 5. Other specify…….. | | | | | | | | | | | |  |  |
| 407 | Did you plan skilled assistant during delivery? | | | | | | | | | | | | | 1. Yes 2. No |  | Q409 |
| 408 | If yes, Whom were you planned to assist you? | | | 1. Physician 2. Health officer 3. Midwife/Nurse 4. HEW 5. Other (specify) | | | | | | | | | | |  |  |
| 409 | During your last childbirth, who was your birth attendant? | | | 1. Health Professional 2. Health extension worker 3. A Traditional birth attendant 4. Relatives 5. If other, explain ----- | | | | | | | | | | |  |  |
| 410 | Had you plan to save money for obstetric emergency? | | | 1. Yes 2. No | | | | | | | | | | |  |  |
| 411 | Did you saved money for obstetric emergency? | | | 1. Yes 2. No | | | | | | | | | | |  |  |
| 412 | Had you plan a mode of transport to place of delivery during emergency? | | | | | | | | 1. Yes 2. No | | | | | |  | Q413 |
| 413 | If yes, What was a mode of transport you had planned?  (ask those planned for emergency transport) | | | 1. On foot 2. By cart 3. On horseback 4. Carried by other people 5. By car 6. Ambulance address 7. Others specify……… | | | | | | | | | | |  |  |
| 414 | Did you plan blood donor during obstetric emergency? | | | 1. Yes 2. No | | | | | | | | | | |  |  |
| 415 | Can you detect early signs of an Emergence? | | | 1. Yes 2. No | | | | | | | | | | |  |  |
| 416 | Did you encounter any health problems during labour, delivery and immediately after birth during your last delivery? | | | | | | | | | | | | 1. Yes 2. No | |  | Q420 |
| 417 | If yes, what were the problems? more than one answer is possible) | | | 1. Excessive vaginal bleeding 2. Prolonged labour(> 12hrs) 3. Retained placenta(>1hrs) 4. Mal-presentation 5. Fetal death 6. Early rupture of membrane 7. Loss of consciousness 8. Other specify | | | | | | | | | | |  |  |
| 418 | Were you referred to HF further? (ask those who faced the problem) | | | | | | | | | | | | | 1. Yes 2. No |  |  |
| 419 | If you were referred to HF, who accompanied you to HF(ask referred) | | | 1. Husband 2. Relatives 3. Community emergency committee 4. Alone 5. Others specify… | | | | | | | | | | |  |  |
| 420 | If you were referred to health facility, what mode of transport you used to reach to the health facility?(ask referred) | | | 1. On foot 2. By cart 3. On horseback 4. Carried by other people 5. By car 6. Ambulance 7. Others specify……… | | | | | | | | | | |  |  |
| 421 | Do you know the availability of free ambulance transport providing service for mothers who are in labour or who encounter pregnancy related problems, if they call the telephone? | | | 1. Yes I know 2. I don’t know | | | | | | | | | | |  |  |
| 422 | Have you Identified institution with a 24 hr EmOC services | | | 1. Yes 2. No | | | | | | | | | | |  |  |
| **Section IV: - Obstetric danger signs awareness** | | | | | | | | | | | | | | | | |
| S.no | Questions | | | Alternatives/ Choices of responses | | | | | | | | | | | Code | Skip |
| 501 | Are there any obstetric danger signs that can occur during pregnancy, labour and postpartum? | | | | | | | | | | | 1. Yes 2. No | | |  | Q503 |
| 502 | If yes, from where did you hear these danger signs? (more than one answer is possible) | | | 1. Health workers 2. HEWs 3. Community H volunteers/HDA 4. Radio 5. Television 6. Read from books 7. Others | | | | | | | | | | |  |  |
| 503 | Are there any danger signs that can occur during pregnancy? | | | 1. Yes 2. No | | | | | | | | | | |  | Q506 |
| 504 | What are these danger signs?  (Wait them to spontaneously mention some or not) more than one answer is possible) | | | 1. Vaginal bleeding 2. Severe headache 3. Blurred vision 4. Convulsions 5. Swollen hands/face*.* 6. High fever 7. Loss of consciousness 8. Difficulty breathing 9. Severe weakness 10. Severe abdominal pain 11. Accelerated/reduced fetal movement 12. Water breaks without labor 13. Other (Specify)---------------------- | | | | | | | | | | |  |  |
| 505 | In your opinion, could women die from [this problem] any of these problems? | | | 1. Yes 2. No   98. Don’t know | | | | | | | | | | |  |  |
| 506 | Are there any danger signs that can occur during labour or child birth? | | | | | | | | | | 1. Yes 2. No | | | |  | Q509 |
| 507 | What are these danger signs?  (Wait them to spontaneously mention some or not) more than one answer is possible) | | | 1. Severe vaginal bleeding 2. Severe headache 3. Convulsions 4. High fever 5. Loss of consciousness 6. Labor lasting>12hours 7. Placenta not delivered 30 minutes after delivery 8. Other (Specify) | | | | | | | | | | |  |  |
| 508 | In your opinion, could women die from [this problem] any of these problems? | | | 1. Yes 2. No   98. Don’t know | | | | | | | | | | |  |  |
| 509 | Are there any danger signs that can occur during postpartum period? | | | | | | | 1. Yes 2. No | | | | | | |  | Finish |
| 510 | What are danger signs that  Can occur during postpartum?  (Wait them to spontaneously mention some or not) more than one answer is possible | | | 1. Severe vaginal bleeding 2. Severe headache 3. Blurred vision. 4. Convulsions 5. Swollen hands/face 6. High fever 7. Loss of consciousness. 8. Difficulty breathing. 9. Severe weakness 10. Malodorous vaginal discharge 11. Others (specify)__________ | | | | | | | | | | |  |  |
| 511 | In your opinion, could women die from [this problem] any of these problems? | | | 1. Yes 2. No   98. Don’t know | | | | | | | | | | |  |  |

This is all what I want to ask you. Thank you for spending your time and valuable information you gave us. Do you have any question that I can address for you?

________________________________________________________________________

________________________________________________________________________

________________________________________________________________________
